# A SWOT Analysis of the Guidelines on Prevention of HIV/AIDS in Japan in the Context of COVID-19

**DOI:** 10.3390/idr13040087

**Published:** 2021-11-05

**Authors:** Kazuki Shimizu

**Affiliations:** Graduate School of Medicine, Hokkaido University, Kita 15 Jo Nishi 7 Chome, Kita-ku, Sapporo 060-8638, Japan; kshimizu@eis.hokudai.ac.jp; Tel.: +81-11-706-5066

**Keywords:** HIV/AIDS, health policy, policy analysis, prevention, testing, antiretroviral therapy, sexual transmitted diseases, pre-exposure prophylaxis, men who have sex with men

## Abstract

In January 2018, the Minister of Health, Labour and Welfare, Japan, released an amended Guideline on the Prevention of Specified Infectious Diseases on Acquired Immunodeficiency Syndrome (AIDS) to propose measures to control the human immunodeficiency virus (HIV)/AIDS. Content analysis was performed to examine the strengths, weaknesses, opportunities, and threats of the guidelines in the context of the ongoing COVID-19 pandemic, thus aiming to promote discussions on the guideline itself and the national HIV/AIDS strategy in Japan in the years ahead. The strengths included the incorporation of the latest scientific advancements, clarification of high-risk populations, an alignment with measures against sexually transmitted diseases (STDs), and willingness towards international cooperation in the Asia-Pacific region. The weaknesses that were exposed included a lack of explicit targets for controlling and containing HIV/AIDS, insufficient descriptions about pre-exposure prophylaxis (PrEP), and aggregated discussions on HIV/AIDS among foreign residents. Although several opportunities for re-energizing the discussions around HIV/AIDS were recognized, insufficient political will and funding, along with the emergence of the ongoing COVID-19 pandemic, could operate as threats. Addressing barriers that were recognized before 2019 and exposed due to the COVID-19 pandemic, and tackling underlying health inequalities through the concept of social determinants of health will be critical.

## 1. Introduction

Although the annual numbers of newly reported human immunodeficiency virus (HIV) and acquired immunodeficiency syndrome (AIDS) patients have peaked at 1126 in 2008 and 484 in 2013, respectively, followed by a slightly decreasing trend, and the prevalence of HIV remains low in Japan, HIV/AIDS has been a critical public health issue in Japan [1]. Recently, 70% of new infections have been reported as originating through transmission from men who have sex with men (MSM), and around 10–15% of cases are reported among foreign residents [1]. The proportion of HIV transmission among Japanese MSM has generally remained at 70% for over ten years, which is higher than its proportion among foreign residents, at around 50–60% [1].

In Japan, countermeasures against HIV/AIDS were implemented by following the Act on the Prevention of Infectious Diseases and Medical Care for Patients with Infectious Diseases. The guideline that is specific to HIV/AIDS was first launched in 1999 and has been amended three times [2]. The latest guideline, which was firstly revised after the introduction of the Joint United Nations Programme on HIV/AIDS (UNAIDS) cascade of “90-90-90” targets [3], which aimed to ensure that 90% of people living with HIV know their status, 90% of people diagnosed with HIV could receive antiretroviral therapy (ART), and 90% of HIV-infected individuals who take ART could suppress the viral load, has been enforced since January 2018, and the guideline is expected to be revisited for further amendments in 2023 [2]. However, in line with the evolution of the coronavirus disease 2019 (COVID-19) pandemic, healthcare resources have been centralized mainly in response to the pandemic, and advances in the prevention and treatment of other infectious diseases have stagnated. In Japan, public health centers, which have primarily worked as a hub for HIV/AIDS services, were overwhelmed by the COVID-19 pandemic [4,5], and a recent study suggested that a rise in HIV cases was missed in the context of COVID-19 in Japan [6]. To contain the HIV/AIDS epidemic and fully utilize available public health resources in the era of COVID-19, revisiting the overall strategy, analyzing the problems, clarifying the future vision, and reconsidering the action plan through back-casting methodology is critical. However, scant research has addressed this.

Therefore, the primary objective of this study is to examine the strengths, weaknesses, opportunities, and threats of the Guidelines on the Prevention of Specified Infectious Diseases on AIDS in Japan. The guidelines were published in 2018, but the context of the ongoing COVID-19 pandemic has prompted urgent consideration of opportunities and threats that are external to the guidelines; thus, there is a need to promote discussions on the guidelines themselves, to reconsider the national HIV/AIDS strategy in Japan, and to provide policy recommendations to inform the next updates.

## 2. Materials and Methods

To analyze the potential impact of the guidelines, highlight internal strengths (S) and weaknesses (W), and clarify the external opportunities (O) and threats (T), the methodology of a SWOT analysis has been employed in line with previous analysis on the Updated National HIV/AIDS Strategy in the U.S. [7]. This methodology has been widely accepted for the analysis of public health strategies and/or plans [8,9,10] and helps find internal and external factors that need to be addressed.

Two steps were undertaken for the investigation. First, the latest Guidelines on Prevention of Specified Infectious Diseases on AIDS in Japan were identified [11] and analyzed through a content analysis approach, as performed elsewhere [4,12,13,14]. While strengths and weaknesses were internally analyzed and extracted, opportunities and threats that are external to the guidelines were examined by reflecting on the context of the current COVID-19 pandemic in Japan. Then, published literature and official documents were visited to analyze the results and deepen discussions. As this study analyzed secondary datasets that were anonymized in advance and publicly available, and patients and the public were not involved, ethical approval by an institutional review board was not required.

## 3. Results

### 3.1. Strengths

Overall, five strengths were extracted from the analysis. First, the guidelines touched on recent scientific evidence regarding HIV/AIDS, such as treatment as prevention (T as P) and emphasized the importance of early detection and treatment to improve prognosis. In this context, progress in the research of the UNAIDS cascade both at national and regional levels was highlighted, and it was argued that an oral consent process for testing is possible. In addition, the guidelines noted the shift from cure to care of older HIV/AIDS patients. Second, the definition of a risk population was revisited to follow the consensus of key affected populations (KAPs), as defined by international organizations such as the World Health Organization (WHO). Additionally, MSM were recognized as the most prioritized group for consideration, and the healthcare services for foreign residents with HIV/AIDS and cooperation with non-governmental organizations were highlighted.

Third, to raise public awareness and knowledge, the guidelines emphasized the need for targeted health promotion at educational institutions for adolescents, among the MSM community, and at healthcare facilities for healthcare workers. The guidelines emphasized the role of healthcare workers in social engagement and the need to enhance their understanding of HIV/AIDS, as well as the importance of taking standard precautions to mitigate fear and anxiety among healthcare workers who are unfamiliar with HIV/AIDS. Fourth, HIV/AIDS and sexually transmitted diseases (STDs) share similar characteristics in prevention, detection, treatment, and supportive care, and inter-collaboration between countermeasures against HIV/AIDS and STDs has been heavily emphasized. In the guidelines on HIV/AIDS, the shared approaches and challenges, along with the importance of simultaneous testing of HIV and STDs, were discussed. Fifth, the necessity of international collaboration, especially in the Asia-Pacific region, was noted.

### 3.2. Weaknesses

Although the guidelines have several strengths, including mention of the scientific advancements in HIV/AIDS research, they also have several weaknesses. First and foremost, there is a lack of reference to scientific evidence and clearly delineated goals. There is also a lack of reference to the extent to which early detection and treatment have contributed to reducing HIV prevalence. Additionally, the numbers of the UNAIDS cascade on achieving 90-90-90 have not been presented. The risk populations were summarized and presented, but an estimated number of each population was not presented in the data. The slogan of “Undetectable = Untransmissible (U = U)” that has been primarily advocated by the Prevention Access Campaign [15] was missing.

Second, despite the increasing trend and potential usefulness of postal HIV testing as suggested by the high demand among MSM [16], effective use of postal testing was still under discussion and concrete measures were not shown. Third, despite emerging evidence of the effectiveness of pre-exposure prophylaxis (PrEP) and discussions on event-driven PrEP, the guidelines did not touch on PrEP, suggesting that discussion on this topic in Japan has stagnated. Fourth, although the prevalence of HIV/AIDS among foreign residents differs according to their nationalities and/or regions of origin, there was little discussion on this disaggregated consideration.

### 3.3. Opportunities (External to the Guidelines)

First, there has been a re-energized recognition of the importance of early treatment of HIV/AIDS in Japan. The latest guideline on anti-HIV treatment recommends that treatment should begin for all people living with HIV, regardless of their CD4 counts. Second, though very modest among Organisation for Economic Co-operation and Development (OECD) countries, the inclusivity of legal lesbian, gay, bisexual, transgender, and intersex (LGBTI) persons in Japan has been improving [17], and there has been increasing awareness of LGBTI rights. Third, as Japan is facing a super-ageing society, the necessity of long-term, whole-person care was well recognized. This could potentially work as an opportunity to support HIV/AIDS patients in the long run. Fourth, the launch of the sub-committee on AIDS and STDs in December 2016 could assist in advancing the countermeasures against both HIV/AIDS and STDs. The revision of the Guidelines on the Prevention of Specified Infectious Diseases on AIDS and STDs is expected to be simultaneously discussed in the years ahead. Finally, the COVID-19 pandemic has highlighted the comprehensive and critical roles of public health centers in Japan, whose objectives range from community health, water, sanitation, and hygiene (WASH); to maternal and child health; to the control of infectious diseases [18]. Especially with regard to infectious diseases, public health centers are essential sites in the collection of data, to conduct epidemiological investigations, test suspected cases and close contacts, summarize results, and provide information to the public [19].

### 3.4. Threats

One of the biggest threats is the lack of an overall strategy against HIV/AIDS in Japan. The guidelines were published by the Minister of Health, but there has been a lack of sector-wide political will to end HIV/AIDS in Japan. Second, Japan still lacks an individual registration system for people living with HIV/AIDS (PLWHA) [20]. This impedes the measurement of the achievements of the UNAIDS cascade. It also hinders the provision of necessary healthcare services to patients at an appropriate time, ensuring patients adhere to their treatment, and understanding the dynamics of diseases, especially among the high-risk populations.

Finally, the current COVID-19 pandemic could work as a threat to advancing discussions on HIV/AIDS as public health centers were overwhelmed by the COVID-19 pandemic [4,5] and a decrease in HIV testing occurred, which, in turn, decreased the reporting of newly diagnosed HIV patients in Japan [6]. COVID-19 has also clarified the underlying discrimination against infected individuals in Japan [21] and the lack of understanding and support available to them. Without appropriate risk communication and community engagement, this attitude could negatively impact PLWHA. Furthermore, an information gap remains between the public, risk populations, and healthcare workers. Due to the relatively low prevalence of HIV in Japan, only a small number of hospitals and healthcare workers have been tackling HIV in Japan, which might have caused a lack of appropriate knowledge on HIV/AIDS among healthcare workers.

**Figure 1:**
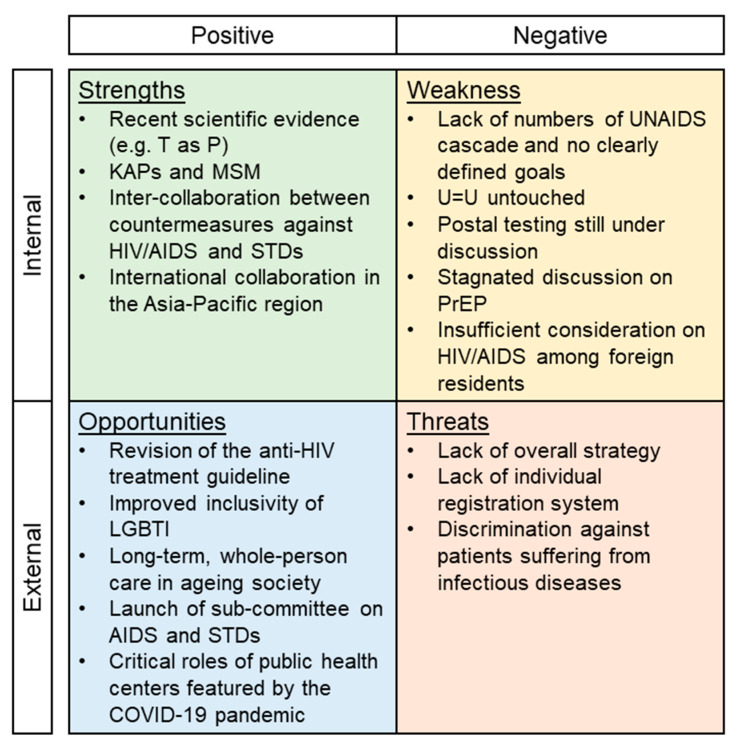
SWOT analysis of the Guidelines on Prevention of HIV/AIDS in Japan.

The results of the analysis on the strengths, weaknesses, threats, and opportunities are summarized below (Figure 1).

## 4. Discussion

This is the first SWOT analysis of the updated Guidelines on the Prevention of Specified Infectious Diseases of AIDS in Japan that reflects on the current COVID-19 pandemic. Several challenges faced in the development of countermeasures against HIV/AIDS were extracted from this study. To consider potential solutions, the discussion will be separated into four pillars.

### 4.1. Prevention

To prevent HIV infection, effective biomedical, behavioral, and structural interventions need to be combined [22]. Highly effective HIV prevention necessitates interventions that are speedy, high quality, large-scale, and sustainable [23]. Real-time analysis of data, urgency of actions to realize lasting benefits, integrated health services based on evidence-informed design, and continuous monitoring and evaluation of data are key [23].

To prevent the transmission of HIV, behavioral change based on the risk profiles of each individual has been emphasized, and the latest guidelines also try to accelerate health promotion campaigns in various settings, such as households, regions, schools, and offices. As PLWHA are still marginalized in the context of Japan, sufficient promotion campaigns that aim to prevent social prejudice and discrimination are vital. Moreover, enhancing the recognition of HIV/AIDS among healthcare workers and enhancing community engagement will be crucial where the HIV prevalence is relatively low and treatment of HIV patients is generally conducted in specific hospitals.

Further, the transmission of HIV has been triggered among MSM through unsafe sexual activities [24]. However, scant discussion has addressed the effective introduction and use of PrEP in the context of preventing HIV transmission in Japan, which differs from the global push to incorporate PrEP in HIV prevention [25]. There has been significant progress around PrEP for previous 10 years, as illustrated by the Iniciativa Profilaxis Pre-Exposición (iPrEx) and Partners PrEP studies [26,27]. Moreover, while it is debatable in its widespread use, the uptake of the event-driven PrEP among MSM [28] needs to be openly discussed. In Japan, a modelling study suggests that the successful introduction of PrEP could contribute to eliminating new HIV infections in Japan [29]. Regrettably, however, the universal health coverage and health insurance systems in Japan do not yet subsidize PrEP, and accessibility is very limited [29]. Considering that combination prevention has become a global consensus to break the chains of HIV transmission [22,23], and scientific evidence supports the introduction of PrEP for high-risk populations in Japan [29], it is expected that more provocative discussions and a process to introduce PrEP could be stipulated in the next updates.

### 4.2. Expanded Testing Opportunities and Antiretroviral Therapy

In Japan, the biggest challenge in achieving the UNAIDS cascade on 90-90-90 by 2020 and 95-95-95 by 2025 has been in the first component: 90% of all people living with HIV will know their HIV status. Previous modelling and epidemiological analysis suggested that the diagnosed proportion of HIV in Japan has not reached 90% [30,31]. Despite this, the guidelines did not explicitly introduce the latest data on the UNAIDS cascade or present concrete objectives. Considering that early diagnosis followed by quick initiation of ART could result in a better prognosis, expansion of testing will be crucial.

Before the emergence of the COVID-19 pandemic, there was already discussion on the issue of access to testing in Japan. Accessibility to sexual health services was limited, especially for MSM [32], and socially discriminatory behaviors among the public towards people living with HIV/AIDS [33] negatively impacted regular HIV testing [32]. Moreover, due to several regulations, it takes a few months for those who test positive for HIV to start ARTs [29].

The COVID-19 pandemic has emerged as a significant threat for PLWHA. Clinically, HIV has been acknowledged as one of significant risk factors for SARS-CoV-2 infection, and the higher mortality by the COVID-19 among PLWHA, which was assumed to be brought by the relatively complicated immune response, was reported, suggesting the necessity of careful consideration to PLWHA [34]. Additionally, from public health viewpoints, the COVID-19 pandemic negatively impacted HIV testing and reporting in Japan [6]. Public health centers, which have played a central role in HIV/AIDS services, have been overwhelmed since the start of the pandemic, and scant discussion has addressed the maintenance of the essential health services that were provided by the public health centers and how these would be continued. As Japan has failed to contain the COVID-19 pandemic [35,36], the impact is expected to last for years. Therefore, outsourcing the role of public health centers in HIV testing, maximizing the effectiveness of postal services, ensuring external quality control, and engaging community centers and PLWHA need to be urgently considered while ramping up efforts for COVID-19 containment.

### 4.3. Leadership, Governance, and Funding

The global AIDS response to date has suggested several key components, such as political leadership, the engagement of civil society and PLWHA themselves, and multi-sectoral collaboration and sector-wide partnerships [22,23]. These core components aim not only to accelerate the containment of the HIV epidemic but also to uphold human rights and address social stigma and discrimination. As highlighted in the newly launched Global AIDS Strategy 2021–2026, the AIDS epidemic will not end without eliminating structural inequalities and addressing social determinants of health [37]. Globally, discrimination originated from the race and unequal gender norms still negatively impact on ensuring their access to education and/or civic participation, bringing challenges in controlling HIV/AIDS in the certain population groups. Considering the existing health inequalities in Japan [38], incorporating the concept of social determinants of health for HIV/AIDS strategy will be an imminent agenda for the next revision.

It should be noted, however, that the leadership and increased political commitment at higher levels to contain HIV and engage civil society and PLWHA are relatively limited in Japan. Unfortunately, there is still no central registry of PLWHA; hence, monitoring and evaluation of links to HIV care from the latest data are impossible. HIV testing at healthcare facilities in outpatient settings is not free, which has resulted in HIV testing hesitancy among both healthcare workers and patients. The lack of data and information could make it challenging to grasp the overall picture of HIV and PLWHA in Japan, and the possibility of double counting has not been eliminated. Therefore, creating a large cohort that is worth monitoring and evaluating for HIV transmission dynamics, sexual behaviors, and number of partners (both heterosexual and homosexual) should be urgently considered. This will help evaluate and achieve the UNAIDS cascade and promote targeted interventions based on risk profiles.

### 4.4. Limitations of This Study

As this study aimed to highlight major points in the current guidelines and incorporate the context of COVID-19, ensuring the comprehensiveness is one of limitations. However, as major challenges are connected to minor issues at sub-national levels, what this study has pointed out could be elaborated in each region, and it is expected that this could be referenced for further discussion.

## 5. Conclusions

This is the first study to analyze the current Guideline on HIV/AIDS by incorporating the perspectives of ongoing COVID-19 pandemic in Japan. To contain HIV/AIDS transmission and achieve the UNAIDS cascade, several weaknesses and threats were analyzed, which were exaggerated by the COVID-19 pandemic. There is an urgent need to address barriers that were recognized before 2019 and exposed due to the COVID-19 pandemic, tackle underlying health inequalities by employing the concept of social determinants of health, and ramp up discussions on the national HIV/AIDS strategy.

## Data Availability

Epidemiological data on HIV/AIDS and the Guidelines are publicly available at https://api-net.jfap.or.jp/status/japan/data/2020/nenpo/2020nenpo.xlsx (accessed on 1 August 2021) and https://www.mhlw.go.jp/file/06-Seisakujouhou-10900000-Kenkoukyoku/0000191837.pdf (accessed on 1 August 2021), respectively.
